# Prediction of Bladder Outlet Obstruction in Male Patients with Lower Urinary Tract Symptoms Based on Symptom Scores and Noninvasive Office-Based Diagnostic Tools

**DOI:** 10.3390/biomedicines13020301

**Published:** 2025-01-26

**Authors:** Min-Ching Liu, Yuan-Hong Jiang, Hann-Chorng Kuo

**Affiliations:** 1 Department of Urology, Hualien Tzu Chi Hospital, Buddhist Tzu Chi Medical Foundation, Tzu Chi University, Hualien 97002, Taiwan; whiteseal1233@gmail.com (M.-C.L.); redeemerhd@gmail.com (Y.-H.J.); 2 Department of Urology, Buddhist Tzu Chi General Hospital, 707, Section 3, Chung Yang Road, Hualien 97002, Taiwan

**Keywords:** bladder outlet obstruction, prostate, symptoms score, lower urinary tract dysfunction

## Abstract

**Purpose:** The purpose of this study was to investigate the predictive values of combining symptom scores, prostatic parameters, uroflowmetry parameters, intravesical prostatic protrusion, and prostatic urethral angle for the establishment of a bladder outlet obstruction (BOO) risk score for diagnosing BOO in men with lower urinary tract symptoms. **Materials and Methods:** A total of 355 men with lower urinary tract symptoms were enrolled and divided into a training set (N = 285) and validation set (N = 70). Videourodynamic studies were used to diagnose lower urinary tract dysfunction subtypes, which were subdivided into a non-BOO and BOO group, which included bladder neck dysfunction and benign prostate obstruction. The parameters were categorized as 0, 1, 2, or 3 according to their cutoff value regarding the specificity of predicting BOO. The BOO risk score was constructed by summing scores of seven variables of symptoms and prostate and uroflowmetry parameters. The area under the curve (AUC) was used to select appropriate cutoff values for predicting lower urinary tract dysfunctions. **Results:** Among the 355 men examined, 234 (65.9%) had BOO, including 136 (38.3%) with bladder neck dysfunction and 98 (27.6%) with benign prostate obstruction. Other lower urinary tract dysfunctions included detrusor overactivity in 37 patients (10.4%), dysfunctional voiding in 28 patients (7.9%), poor relaxation of the external sphincter in 26 patients (7.3%), detrusor underactivity in 14 patients (3.9%), stable bladder in 9 patients (2.5%), and a hypersensitive bladder in 7 patients (2%). With the summation of the BOO risk scores from each variable, a BOO risk score of ≥10 could yield a sensitivity of 0.822 and specificity of 0.656 for benign prostate obstruction in the training set [AUC = 0.800 (0.741–0.859)] and a sensitivity of 0.80 and specificity of 0.64 in the validation set [AUC = 0.813 (0.701–0.925)]. **Conclusions:** With office-based diagnostic tools, including symptom scores and uroflowmetry and prostate parameters, a BOO risk score was established. A BOO risk score of ≥10 can predict the presence of benign prostate obstruction in >80% of men with lower urinary tract symptoms refractory to initial medication.

## 1. Introduction

Lower urinary tract symptoms (LUTSs) are prevalent in men of all ages and represent a challenge when it comes to diagnosing the underlying etiology [1]. Previous studies have revealed that bladder outlet obstruction (BOO) is only present in one-third of men with LUTSs [2]. The pathophysiology of BOO in men involves both anatomical and functional disorders, such as benign prostatic obstruction (BPO), bladder neck dysfunction (BND), dysfunctional voiding (DV), poor relaxation of the external sphincter (PRES), and urethral stricture [2,3,4]. Additionally, in men with LUTSs, common lower urinary tract dysfunctions (LUTDs) include a hypersensitive bladder, detrusor overactivity (DO), and detrusor underactivity (DU), even after medical or surgical management of BOO [2,5,6]. A differential diagnosis based on symptoms alone is unreliable; however, adding a larger prostatic volume and lower maximum flow rate to form a nomogram may increase the accuracy of BOO diagnoses in men [7].

Determining the subtypes of LUTDs associated with BOO in men is pivotal for choosing the optimal medication and correct management. [8] Previous studies involving men with LUTSs have revealed that LUTSs alone cannot provide accurate diagnoses of LUTDs [9]. Videourodynamic studies (VUDSs) enable a more precise and comprehensive examination of bladder and bladder outlet conditions in both the storage and emptying phases [2]. VUDS record real-time data of pressure–flow studies and concomitant fluoroscopic imaging during both the storage and emptying phases of the bladder, providing an understanding of the underlying anatomical dysfunction in LUTD patients [10]. However, VUDSs have disadvantages, such as invasiveness and complicated protocols. Thus, VUDSs are not routinely used for patients with LUTSs but are only employed when initial medication has failed to resolve LUTSs or invasive surgery is planned [11].

First-line examinations of LUTDs in men with LUTSs include the International Prostate Symptom Score (IPSS), voiding-to-storage (V/S) ratio, prostatic parameters, uroflowmetry parameters, and prostate-specific antigens, which are mainly used to diagnose BPO [12]. Cystoscopy or urodynamic studies are usually performed when the treatment based on the initial diagnosis is not effective. Combinations of several office-based examinations that achieve an accurate diagnosis of BPO have been reported [7,13]. However, a single examination might not accurately predict the presence of BOO, including BND, BPO, PRES, or DV in males with LUTSs. Using VUDSs, the precise LUTD can be detected and effective treatment be provided [11]. VUDSs are an expensive way to achieve a differential diagnosis of male LUTSs and are not applicable in most clinics; therefore, we aimed to construct a BOO risk score by means of office-based investigative tools such as symptom scores, uroflowmetry parameters, and prostate variables.

To the best of our knowledge, no study to date has developed prediction models using VUDSs as the final diagnostic standard, especially for men with suspected medication-refractory BOO. This study investigated the predictive values of BOO risk scores in diagnosing BPO, BND, and other LUTDs in men with LUTSs refractory to initial medications. The results of this study can provide a reference for clinicians to select the appropriate medication and management for BPO in male patients.

## 2. Materials and Methods

### 2.1. Patients

This retrospective study included a cohort of male patients with medication-refractory, nonneurogenic LUTSs and suspected BOO. All patients underwent VUDSs prior to enrollment at a single medical center from January 2016 to March 2023. The inclusion criteria included men who had been on continuous medication, e.g., alpha-blockers for voiding LUTSs and antimuscarinics for storage LUTSs, for more than 3 months without a satisfactory response. The patients were recommended to have discontinued previous medication for more than 1 month before proceeding to VUDS. Exclusion criteria included patients that were under 20 years of age; presented with overt neurogenic LUTDs; had acute or chronic urinary retention due to an enlarged prostate (more than 60 mL); had a bladder stone or gross hematuria complicated with overt BOO; had medical comorbidities such as insulin-dependent diabetes mellitus, chronic obstructive pulmonary disease, coronary heart diseases, congestive heart failure, chronic kidney diseases, or other chronic debilitative diseases; had previous lower urinary tract surgery, had any history of urological malignancy by chart review, or lacked interpretable VUDS records.

This study was approved by the Institution Review Board of the hospital (IRB 111-132-B). Informed consent was waived due to the retrospective nature of the analysis. The study was conducted in accordance with the ethical principles of the Declaration of Helsinki from 2013.

### 2.2. Noninvasive Examinations and VUDS

For all male patients with LUTSs, we routinely recorded their history, their physical examinations, digital rectal examination of the prostate, IPSS subscores, V/S ratio, serum prostate-specific antigen levels, and transrectal ultrasound of the prostate, along with uroflowmetry. The noninvasive clinical parameters used to construct the predictive model were derived from these office examinations. The parameters were categorized as 0, 1, 2, or 3, according to their cutoff values with regard to the specificity of predicting BOO. The BOO risk score was constructed by summing scores of seven variables of symptoms, prostate parameters, and uroflowmetry parameters. The measured parameters included maximum flow rate (Qmax), voided volume, and postvoid residual (PVR) volume. IPSS subscores included IPSS-voiding, IPSS-storage, and IPSS-V/S ratio [14]. Transrectal ultrasound of the prostate measurements included the total prostate volume (TPV), transitional zone index (TZI, defined as the percentage of transitional zone volume to TPV), intravesical prostatic protrusion (IPP, measured in cm), and prostatic urethrovesical (PUV) angle (measured in degrees) [15].

VUDSs were performed on patients in the standing position, as previously reported [16]. The VUDSs’ parameters included the first sensation of bladder filling, full sensation, urge sensation, bladder compliance, voiding detrusor pressure at Qmax (Pdet.Qmax), Qmax, corrected Qmax (cQmax, defined as Qmax divided by the square root of the bladder volume), voided volume, PVR (measured using bladder sonography), and BOO index. All descriptions and terminologies used in the VUDS were in accordance with the recommendations of the International Continence Society [17,18].

DO was defined as urodynamic evidence of spontaneous detrusor contractions occurring during bladder filling or before uninhibited detrusor contraction voiding upon reaching bladder capacity. DU was defined as having a voiding detrusor contractility of <10 cmH_2_O, having the need to void by abdominal straining, or being unable to void. DO with DU (DO-DU) was defined as DO associated with incomplete bladder emptying and a PVR of >100 mL [18]. BOO was defined by radiographic evidence of narrowing in voiding cystourethrography at the bladder neck (defined as BND), prostatic urethra (defined as BPO), urethral sphincter, or distal urethra (defined as urethral stricture), with a sustained detrusor contraction of high or low magnitude [5,8,19]. The diagnosis of BND was made based on a narrow bladder neck and open prostatic urethra during voiding with or without an elevated Pdet during voiding [20]. BPO was diagnosed when the prostatic urethra remained narrow with or without a narrow bladder neck and Pdet was elevated during voiding [2,5]. Patients were diagnosed with PRES without true BOO when they exhibited a low Pdet.Qmax, low Qmax, an open bladder neck and prostatic urethra, and a narrow urethral sphincter in the VUDS [5]. Patients without VUDS-determined BOO were considered non-BOO. All VUDS reports were collected and reviewed by a single urology professor to ensure consistency and reliability in the interpretation of these complex studies.

### 2.3. Outcome Measures and Statistical Analysis

Several outcome measures were used to evaluate the effectiveness of our predictive models. Discrimination (the model’s ability to distinguish between the presence and absence of an outcome) was measured by the area under the receiver operating characteristic curve (AUC). The AUC provides a single measurement that summarizes the model’s accuracy across all classification thresholds. The characteristic variables for BOO risk score were obtained from significant difference variable between BOO and non-BOO patients. The numerical risk score was established by the predictability of BOO based on the reported data [6,7,13,14,15,21,22]. Optimal BOO risk scores for diagnosing BPO, BND, and other LUTDs were identified from the receiver operating characteristic curves using Youden methods. The sensitivity and specificity of diagnosing BOO with the proposed BOO risk scores were also examined in patients with VUDS-determined BPO, BND, or other LUTDs.

Categorical variables are presented as the number of patients (proportion), and continuous variables are presented as means ± standard deviations. A *p* value of <0.05 was considered statistically significant. All statistical analyses were performed using Statistical Package for the Social Sciences software for Windows (version 25.0; SPSS, Chicago, IL, USA).

## 3. Results

### Patient Characteristics and VUDS Diagnosis

This cohort included 355 male patients presenting with medication-refractory LUTSs and suspected BOO, all of whom underwent VUDS before enrollment. Patients with a TPV of >60 mL and acute or chronic urinary retention were not included in this study. The cohort was divided into a training set (80%, n = 285) and a validation set (20%, n = 70). The mean age of the entire cohort was 67.8 ± 9.5 years, which did not significantly differ between the training and validation sets (*p* = 0.464). Comparisons of the training and validation sets revealed no significant differences in the following clinical parameters: IPSS-voiding, IPSS-storage, IPSS-V/S ratio, Qmax, voided volume, PVR, TPV, TZI, IPP, and PUV angle (Table 1).

The VUDS diagnoses revealed obstruction-predominant conditions, with BND accounting for 38.3% (n = 136), BPO for 27.6% (n = 98), DV for 7.9% (n = 28), and PRES for 7.3% (n = 26) of cases. Regarding bladder dysfunction, DU without BOO was present in 3.9% (n = 14), DO without BOO in 10.4% (n = 37), hypersensitive bladder in 2% (n = 7), and a normal bladder in 2.5% (n = 9) of cases. The distributions of these LUTDs were similar between the training and validation sets (Table 2).

Table 3 shows the clinical symptoms and uroflowmetry and prostate parameters of patients with and without BOO. BOO patients exhibited significantly older age, lower Qmax values, and higher values of TPV, TZI, IPP, and PUV angles.

Based on the sensitivity and specificity of each single variable for BOO, a clinical BOO risk scoring system was established, using the measured parameters obtained from noninvasive examinations, including age, IPSS-V/S, uroflowmetry parameters (Qmax and voided volume), and prostate parameters (TPV, TZI, IPP, and PUV angle). The variables were categorized as 0, 1, 2, or 3 according to their cutoff values with regard to predicting BOO (Table 4).

Table 5 shows the sensitivity, specificity, and AUC of the training and validation sets of patients using the BOO risk score. The predictive model showed that for a BOO risk score of ≥10, the sensitivity and specificity for BPO were 82.2% and 65.6%, respectively, in the training set and 80.0% and 64.0%, respectively, in the validation set. The AUC was 0.800 (confidence interval: 0.741–0.859) for the training set and 0.813 (confidence interval: 0.701–0.925) for the diagnosis of BPO by a BOO risk score of ≥10. The diagnostic accuracy of the other variables was lower than 0.700 using the BOO risk score system. Based on these results, a BOO risk score of ≥10 can predict the presence of BPO in >80% of men with LUTSs.

## 4. Discussion

This study established a BOO risk score system based on office-based examinations, including IPSS, uroflowmetry, and prostate parameters. A BOO risk score of ≥10 can predict the presence of BPO in >80% of men with LUTSs. However, patients with a BOO risk score of <10 may need to undergo comprehensive VUDSs to identify the LUTD subtype. This study highlights the use of VUDSs to identify LUTDs in men. Most previous studies only used pressure–flow studies as the standard method for assessing BOO [7,21]. Using the BOO risk score, a predictive model based on office-obtained parameters can be established.

Previous research on factors for predicting BOO in men with LUTSs has yielded variable results. Chia et al. reported that the diagnostic accuracy for an IPP of >10 mm had a specificity of 92%, which was similar to the result obtained when a Qmax of <10 mL/s was used for free-flow rate testing [22]. Cicione et al. presented a multivariable logistic age-adjusted regression model, which revealed that bladder wall thickness, PVR, and TPV were significant predictors for BOO. The model had an accuracy of 0.82 and a clinical net benefit of 10–90% [23]. However, the diagnosis of BOO was based on pressure–flow studies and not VUDSs. Therefore, patients with BND and DV might be diagnosed with BOO due to prostatic obstruction and might then require transurethral prostatic surgery. This study revealed that BPO was detected in 27.6% of men with LUTSs based on the VUDS findings, whereas BND was detected in 38.3% and DV in 7.9%. With the BOO risk score, the diagnosis accuracy of BPO can be increased to more than 80%, further addressing that combining multiple variables can provide precision diagnosis of BPO and other LUTDS.

Several noninvasive techniques have also been developed for the assessment of BOO in men with LUTSs. From the evidence reviewed by Malde et al., penile cuff tests have a sensitivity of 88.9% and specificity of 75.7%, with positive and negative predictive values of 67.7% and 93.0%, respectively [24]. Detrusor wall thickness and IPP by suprapubic ultrasound have a high predictive value for BPO in men [25]. Near-infrared spectroscopy can identify men with BOO with a sensitivity of 68.3%, specificity of 62.5%, and average diagnosis coincidence rate of 66.7%, compared with the gold standard—the Abrams-Griffiths number [26]. These studies all showed high diagnostic accuracy for BOO in men. However, the diagnoses of BOO and BPO in these studies were not based on VUDSs, and thus may have resulted in bias regarding the final definition of BOO and surgical plans. Current guidelines of male LUTSs recommend that invasive surgical procedure should be performed only when a precision diagnosis of BPO has been established [11,12].

It is likely that no single test is universally suitable for diagnosing all patients with LUTSs and suspected BOO. All simple tests, aside from VUDSs, have exhibited only limited diagnostic accuracy for BOO, and no single test can definitively identify BND or BPO [27]. Urination is a dynamic process involving bladder contractions and bladder outlet relaxation, and therefore static measurement of the TPV, TZI, or PUV angle might not accurately reflect the voiding condition during urination [2,5,10]. Therefore, it is necessary to combine several parameters from simple tests to achieve satisfactory diagnostic accuracy that is similar to that obtained using VUDS. Kuo has proposed a clinical prostate score in patients with LUTSs suggestive of BPH. Parameters with a positive prediction of LUTSs due to BPH were scored +1 or +2, whereas those with a negative prediction scored −1 or 0. The sensitivity and specificity of BPO diagnoses in patients with at least one favorable predictive factor with a total score of ≥3 were 91.6% and 87.3%, respectively [28]. However, in that study, the author did not divide BOO into BPO and BND.

The BOO in men with LUTSs included not only BPO, but also BND and DV. The patients with BND and DV might have small prostatic volume but inadequate relaxation of the bladder neck or urethral sphincter during voiding [2]. Transurethral resection of the prostate for patients with BND and DV is not appropriate and could increase the risk of postoperative complications. Although a larger TPV indicates higher incidence of BPO in male patients with LUTSs, this study shows that for 46.2% of patients with LUTSs and BOO, their conditions are actually a result of BND or DV, especially in patients with a TPV of <40 mL. Our current study did not include patients with a TPV of >60 mL and acute or chronic urinary retention. Therefore, the sensitivity and specificity of BPO were lower than those in the previous study [28]. However, we used VUDS to identify BND, PRES, and other LUTDs, and the results of this study reflect real-life practices in diagnosing BPO.

This study investigated patients with voiding LUTSs and moderately enlarged prostates who were refractory to initial medical treatment. Although all patients had LUTSs, the LUTDs varied widely and included BOO (BND, BPO, DV, PRES) and bladder dysfunction (stable bladder—DO; hypersensitive bladder—DU). These results reveal that LUTSs in men cannot be reliably diagnosed with a single test, and thus combining parameters from several noninvasive tests may have a more accurate predictive value [9,27]. However, using numerous variables to create a scoring system for predicting BOO may not result in a high predictive value because BOO (diagnosed with VUDSs) may be accompanied with BPO, BND, DV, and possible PRES. In this study, the scoring system used to predict BPO was satisfactory; approximately 80% of BPO patients could be identified with a BOO risk score of ≥10. However, the specificity was 65%, which is not sufficient for a clinically useful scoring system for predicting BPO for invasive treatment. The results of this study indicate that an accurate diagnosis of BOO subtypes in men cannot rely on a single office-based examination. When the diagnosis of BPO is not definite, VUDS may be necessary to avoid incorrect diagnosis or overtreatment [2,16].

Several medical diseases may contribute to the development of LUTSs in elderly men, including insulin-dependent diabetes mellitus, chronic obstructive pulmonary disease, coronary heart diseases, congestive heart failure, chronic kidney diseases, or other chronic debilitative diseases. Obstructive sleep apnea syndrome is often overlooked in evaluation of male LUTSs like nocturia and nocturnal polyuria [29]. Male patients may have one of these medical comorbidities that cause the LUTSs to mimic BOO. Therefore, the initial medication targeting at BPO or BND fails to improve LUTSs. This study used VUDSs to evaluate the diagnostic accuracy of BOO risk score on BND, BPO, and other LUTDs, further addressed the strength of combining multiple office-based noninvasive urological examination variables in the diagnosis of BPO.

This study has collected several noninvasive parameters obtained from office-based examinations that show promise for assessing BPO in men with LUTSs suggestive of BOO. However, several limitations should be reported. First, this is a retrospective analysis, which introduces a potential selection bias due to the inclusion of only those patients who had LUTSs refractory to the initial medical treatment, and the exclusion of patients with a large TPV, urinary retention, and incomplete data. Therefore, patients with BND or BPO who were responsive to the initial medical treatment were not included, which might have decreased the sensitivity and specificity of this BOO risk score in diagnosis of these LUTDs. Second, the study used data from only one medical center, which might limit the generalizability of the findings. For further validation, multicenter data should be pooled together to enhance the robustness of our predictive model. Furthermore, this BOO risk score system was constructed to predict BOO in male patients with LUTSs who did not respond to the initial medication. Our developed model might not be suitable for patients who require initial diagnoses of LUTDs.

## 5. Conclusions

With office-based urological examinations, including IPSS, uroflowmetry, and prostate measurements, a BOO risk score can be established. A BOO risk score of ≥10 can predict the presence of BPO in >80% of men with LUTSs. This study demonstrated the accuracy of predicting BPO in men with LUTSs, which can be used to guide subsequent treatment or invasive examinations. However, for patients with a BOO risk score of <10, VUDSs are still needed for an accurate diagnosis of BND, PRES, or other LUTDs in men with LUTSs refractory to initial medical treatment.

## Figures and Tables

**Table 1:** The symptom scores and prostatic and uroflowmetry parameters of the training and validation sets.

Characteristics	Total Patients 355 (100%)	Training Set 285 (80.3%)	Validation Set 70 (19.7%)	*p* Value
Age	67.8 ± 9.5	67.9 ± 9.6	67.0 ± 9.1	0.464
IPSS-Voiding	9.5 ± 5.8	9.7 ± 5.8	8.8 ± 5.7	0.241
IPSS-Storage	8.6 ± 4.1	8.6 ± 4.1	8.3 ± 4.2	0.583
IPSS-V/S	1.5 ± 1.7	1.5 ± 1.7	1.4 ± 1.7	0.450
Qmax (mL/s)	10.4 ± 6.5	10.6 ± 6.7	9.8 ± 5.8	0.362
VoL (mL)	197.8 ± 123.3	200.3 ± 127.4	187.6 ± 105.4	0.438
PVR (mL)	46.9 ± 86.9	46.5 ± 77.4	48.3 ± 118.7	0.876
TPV (mL)	37.3 ± 19.0	36.9 ± 18.5	39.3 ± 21.0	0.341
TZI	0.40 ± 0.16	0.40 ± 0.16	0.42 ± 0.16	0.195
IPP	0.3 ± 0.6	0.3 ± 0.6	0.3 ± 0.6	0.663
PUV angle	26.9 ± 18.8	26.6 ± 18.8	27.9 ± 19.0	0.621

IPSS: International Prostate Symptom Score, V/S: voiding to storage ratio, Qmax: maximum flow rate, Vol: voided volume, PVR: post-void residual, TPV: total prostate volume, TZI: transition zone index, IPP: intravesical prostatic protrusion, PUV: prostatic urethrovesical.

**Table 2:** The number of patients with LUTDs determined by videourodynamic studies (VUDSs).

VUDS Diagnosis	Total Patients (N = 355)	Training Set (N = 285)	Validation Set (N = 70)
Bladder neck dysfunction	136 (38.3%)	111 (38.9%)	25 (35.7%)
Benign prostate obstruction	98 (27.6%)	73 (25.6%)	25 (35.7%)
Poor relaxation of external sphincter	26 (7.3%)	23 (8.1%)	3 (4.3%)
Dysfunctional voiding	28 (7.9%)	23 (8.1%)	5 (7.1%)
Stable bladder without BOO	9 (2.5%)	6 (2.1%)	3 (4.3%)
DO without BOO	37 (10.4%)	31 (10.9%)	6 (8.6%)
Hypersensitivity without BOO	7 (2.0%)	5 (1.8%)	2 (2.9%)
Detrusor underactivity	14 (3.9%)	13 (4.6%)	1 (1.4%)

BOO: bladder outlet obstruction.

**Table 3:** The symptoms and prostatic and uroflowmetry parameters of patients with and without BOO.

Characteristics Variables	Total Patients (N = 355)	BOO (N = 234)	Non-BOO (N = 121)	*p*-Value
Age (years)	67.8 ± 9.5	68.6 ± 8.8	66.2 ± 10.7	0.037
IPSS-Voiding	9.5 ± 5.8	9.1 ± 5.7	10.1 ± 6.0	0.120
IPSS-Storage	8.6 ± 4.1	8.5 ± 4.1	8.7 ± 4.0	0.705
IPSS-V/S	1.5 ± 1.7	1.5 ± 1.7	1.6 ± 1.7	0.483
Qmax (mL/s)	10.4 ± 6.5	9.4 ± 5.5	12.4 ± 7.7	<0.001
Voided volume (mL)	197.8 ± 123.3	192.3 ± 120.5	208.4 ± 128.3	0.244
PVR (mL)	46.9 ± 86.9	52.4 ± 89.0	36.2 ± 82.0	0.095
TPV (mL)	37.3 ± 19.0	40.7 ± 20.5	30.8 ± 13.4	<0.001
TZI	0.40 ± 0.16	0.43 ± 0.15	0.35 ± 0.15	<0.001
IPP (cm)	0.3 ± 0.6	0.4 ± 0.6	0.1 ± 0.4	<0.001
PUV angle (degree)	26.9 ± 18.8	31.1 ± 17.9	18.6 ± 17.8	<0.001

BOO: bladder outlet obstruction, IPSS: International Prostate Symptom Score, V/S: voiding to storage ratio, Qmax: maximum flow rate, Vol: voided volume, PVR: post-void residual, TPV: total prostate volume, TZI: transition zone index, IPP: intravesical prostatic protrusion, PUV angle: prostatic urethrovesical angle.

**Table 4:** The BOO risk scores defined according to the sensitivity and specificity of each measured variable in men with LUTSs.

Variables	Cut Off Value	Diagnosis of BOO		Risk Score
		Sensitivity	Specificity	
Age (years)	<65 ≥65	70.7%	43.6%	0 1
IPSS-V/S	<1.0 1.0–1.9 ≥2.0	56.0% 19.0%	34.7% 69.3%	0 1 2
Qmax (mL/s)	>15 13–15 11–12 ≤10	85.3% 72.3% 63.6%	24.8% 35.6% 47.5%	0 1 2 3
Voided volume (mL)	>300 201–300 151–200 ≤150	81.5% 58.7% 41.8%	23.8% 46.5% 62.4%	0 1 2 3
TPV (mL)	<30 30.0–39.9 40.0–49.9 ≥50	64.7% 39.7% 26.1%	57.4% 85.1% 92.1%	0 1 2 3
TZI	≤0.30 0.31–0.49 ≥0.50	80.4% 29.9%	31.7% 82.2%	0 1 2
IPP (cm)	<0.5 0.5–0.9 ≥1.0	37.5% 25.5%	90.1 95.0%	0 1 2
PUV angle (degree)	<30 30–39 ≥40	69.6% 38.0%	60.4% 83.2%	0 1 2
Total score				0–20

BOO: bladder outlet obstruction, IPSS: International Prostate Symptom Score, V/S: voiding to storage ratio, Qmax: maximum flow rate, Vol: voided volume, PVR: post-void residual, TPV: total prostate volume, TZI: transition zone index, IPP: intravesical prostatic protrusion, PUV angle: prostatic urethrovesical angle.

**Table 5:** The predictive values of BPO and other LUTDs according to the BOO risk scores.

	BOO	Training Set (N = 285)			Validating Set (N = 70)		
	Risk Score	Sensitivity	Specificity	AUC (95% CI)	Sensitivity	Specificity	AUC (95% CI)
BPO	≥10	0.822	0.656	0.800 (0.741–0.859)	0.80	0.64	0.813 (0.701–0.925)
BND	≤7	0.405	0.736	0.578 (0.512–0.645)	0.32	0.71	0.596 (0.464–0.728)
PRES	≤8	0.652	0.573	0.619 (0.489–0.749)	0.33	0.60	0.443 (0.117–0.768)
DV	≤9	0.739	0.485	0.611 (0.508–0.714)	0.60	0.52	0.712 (0.496–0.928)
DU	≤11	0.846	0.279	0.513 (0.361–0.665)	1.00	0.30	0.558 (0.434–0.682)
HSB and Normal	≤9	0.818	0.478	0.685 (0.551–0.819)	0.60	0.52	0.651 (0.458–0.843)

BOO: bladder outlet obstruction; AUC: area under curve; CI: confidence interval; BPO: benign prostatic obstruction; BND: bladder neck dysfunction; PRES: poor relaxation of external sphincter; DV: dysfunctional voiding; DU: detrusor underactivity; HSB: hypersensitive bladder.

## Data Availability

The original contributions presented in this study are included in the article. Further inquiries can be directed to the corresponding author.
